# The centrality of nursing in realizing high quality palliative care: Exploring Canada’s framework on palliative care priorities

**DOI:** 10.1186/s12912-024-02488-6

**Published:** 2024-11-08

**Authors:** Barbara Pesut, Sally Thorne, David Kenneth Wright, Michael Banwell

**Affiliations:** 1 University of British Columbia, 1147 Research Way, 3rd Floor Arts Building, Kelowna, V1V 1V7 BC Canada; 2 University of British Columbia School of Nursing, T213 2211 Wesbrook Mall, Vancouver, BC V6T 2B5 Canada; 3 School of Nursing, University of Ottawa, 75 Laurier Ave, East, Ottawa, ON K1N 6N5 Canada

**Keywords:** Canada, End of life, Palliative care, Qualitative, Interpretive description, Interviews, Quality improvement, Policy

## Abstract

**Background:**

Following an earlier mixed-method survey in which we asked stakeholders to report on their perceptions of the progress made in relation to Canada’s Framework on Palliative Care and Action Plan, the purpose of this study was to conduct an in-depth qualitative exploration of the factors influencing that progress, or lack thereof.

**Methods:**

This was a qualitative interview study conducted in Canada. Inclusion criteria included experience with palliative care in Canada in a professional or volunteer capacity. Interviews were conducted by telephone using an interview guide that asked specific questions in relation to the Framework on palliative care priorities (e.g., education, caregiver support, and equitable access). Data was analyzed using qualitative descriptive methods.

**Results:**

Thirty-five diverse stakeholders with extensive experience in palliative care were interviewed. In relation to palliative education, participants indicated that although there were excellent palliative care resources available across the country there was further need for embedding palliative care in undergraduate education and for mentored opportunities to engage in care across diverse contexts. The identification, development, and strategic positioning of champions was an important strategy for improving palliative care knowledge and capacity. The development of standard competencies was viewed as an important step forward; although, there was a need to include more members of the care-team and to create pathways for life-long learning. In relation to support for family caregivers, even as participants cited numerous community-based resources offered by not-for-profit organizations, they described significant barriers including a shortage of in-home support, lack of understanding of what caregivers do, and policy-based contractual and privacy issues. In relation to palliative care access, participants described a nurse-centered, consult-based, multi-site and multi-provider model of care that was facilitated by technology. Barriers to this model were systemic healthcare issues of siloed, fragmented, and for-profit care.

**Conclusion:**

Participants in this study had clear insights into the factors that would support or impede progress to the development of palliative care in Canada. Some of those factors were achievable within current health and educational systems. Other factors were going to require longer term and more comprehensive solutions.

## Background

Between 1995 and 2015, there were no less than 18 national reports with recommendations for improving palliative care in Canada [1]. However, determining progress on those recommendations has been challenging because of the lack of good data about palliative care in Canada [2]. Based on the limited data available, a 2023 report by the Canadian Institute for Health information indicated that although more people over the past five years are receiving palliative care and dying at home, there are still indicators of poor palliative care overall and specific barriers to access to palliative care related to age, geographic area of residence, and disease diagnosis [2]. On a global level, in a comparison of 81 countries, Canada ranked 22nd on performance related to quality of death and dying [3]. 

There are several factors that make the delivery of high quality palliative care challenging in Canada. Canada’s extensive rural geography creates unique challenges for providing high quality healthcare, including palliative care, to rural citizens. Further, responsibility for delivering healthcare lies with the 10 provincial and 3 territorial governments rather than at the federal level. This can make it challenging to negotiate common national priorities around common concerns, and historically, Canada has not had national laws, accountabilities and deliverables for palliative care. In the 2019 European Association Atlas of Palliative Care, of the 54 countries surveyed, 76% had adopted laws to include palliative care as a mandatory service or human right, and 35 of 51 countries reported having a key leadership position in the government dedicated to palliative care policies [4]. Only recently (2017) has Canada taken a similar approach.

An important strategy to improving palliative care across the provinces and territories of Canada has been the formation of the Palliative Care Coalition of Canada that includes over 36 partner organizations [5]. In a recent submission to the Special Joint Committee on Medical Assistance in Dying [6], this coalition identified two key weaknesses in palliative care. First, Canada relies too heavily on private funding in that important aspects of care are delivered through the voluntary sector and patients and families incur substantial out of pocket expenses. Second, there are particular populations at risk for poor palliative care, including those experiencing homelessness, those living in rural and remote communities, and Indigenous persons.

Despite these historic limitations, Canada has now made important progress in a national approach to palliative care. In 2017, the federal government passed a Bill calling for a national framework on palliative care [7]. The *Framework on Palliative Care in Canada Act* outlined as a priority goal the improvement of access to palliative care for Canadians both within community and also in institutions such as long term care and residential hospice. The *Act* articulated its explicit approaches to meeting this goal as a framework that: “defines what palliative care is; identifies the palliative care training and education needs of healthcare providers as well as other caregivers; identifies measures to support palliative care providers; promotes research and the collection of data on palliative care; identifies measures to facilitate a consistent access to palliative care across Canada; takes into consideration existing palliative care frameworks, strategies and best practices; and evaluates the advisability of re-establishing the Department of Health’s Secretariat on Palliative and End-of-Life Care” (para 2) These goals formed the guiding direction for a subsequent Framework on Palliative Care in Canada [which we will refer to as “the Framework”] in 2018 [8] and Action Plan on Palliative Care [which we will refer to as “the Action Plan”] in 2019 [9]. 

Using the Framework as a guide, our purpose in this study was to explore the perceptions of Canadian palliative care stakeholders about the progress in palliative care since the establishment of the Framework in 2017. We conducted an online mixed-method survey designed to evaluate improvements across five domains and 29 items based upon the Framework. The quantitative survey results, which were published previously, indicated that palliative stakeholders felt the *most improvement* had occurred in palliative care education, advance care planning and the use of technology [10]. The *least improvement* had occurred in support for family caregivers, bereavement services, and in-home support [10]. At the conclusion of that survey, respondents were invited to participate in an interview to further share their experiences of providing palliative care in the Canadian context. The goal of these interviews was to provide a more nuanced understanding of data obtained from the survey and to query study participants about specific innovations. This paper reports on the findings from analysis of those subsequent interviews.

## Methods

### Aim, Design, Setting

The aim of this qualitative interview study was to explore stakeholder perceptions of the progress in palliative care in Canada. The research question was: *What do participants see as the most significant developments*,* gaps*,* and barriers to achieving high quality palliative care in relation to the national Framework on Palliative Care priorities?*

### Participants

Inclusion criteria included experience with palliative care in Canada in a professional or volunteer capacity. Participants were recruited through an online survey; 35 of 150 survey participants agreed to be interviewed. Thirty-five persons from across Canada participated in an interview. The sample included representation from diverse stakeholders with extensive palliative care experience (See Table 1).

**Table 1:** Demographic Profile of Participants (*n* = 35) Each variable should be highlighted similar to “professional background” Otherwise the table is too difficult to read

Professional Background	N (%)
Nurse	13 (37.1)
Other (Chaplin, Executive Directors, Leadership. Doula)	11 (28.6)
Physician	5 (14.3)
None	3 (8.6)
Nurse Practitioner	2 (5.7)
Social Worker	1 (2.9)
Primary role in palliative care (one or more answers possible)	
Specialized palliative care provider (general population)	15 (42.8)
Other (Educators, Executive Directors, Spiritual Care, Doula)	9 (25.7)
Volunteer	5(14.3)
Non-specialized palliative care provider (e.g., community health nurse, family physician)	5(14.3)
System level decision-maker/leader for palliative care or palliative approach to care	3(8.6)
Researcher	3 (8.6)
Education in specialized palliative care or in a palliative approach to care	
Yes	28 (80.0)
No	4 (11.4)
Unsure	3 (8.6)
# Years working/volunteering in palliative care	
1–4	6 (17.1)
5–9	9(25.7)
10–14	6 (17.1)
15–19	3 (8.6)
> 20	11 (31.4)
Province/ Territory of Work	
BC	9 (25.7)
Alberta	4 (11.4)
Saskatchewan	1 (2.9)
Manitoba	1 (2.9)
Ontario	12 (34.3)
Quebec	2 (5.7)
Nova Scotia	3 (8.6)
New Brunswick	3 (8.6)
Nature of Geographic Location (one or more answers possible)	
Urban (> 100,000)	17 (48.6)
Small urban (10,000 to 99,000 population)	11 (31.4)
Rural (< 10,000 population)	3 (8.6)
Across all contexts	4 (11.4)

### Data collection and analysis

Telephone interviews were conducted between December 2021 and February 2022 by BP and a research coordinator (RC) using an interview guide that had been previously piloted and that asked specific questions on palliative care priorities in relation to the national Framework (e.g., education, caregiver support, and equitable access) (See Table 2). Interviews lasted 30 to 60 min and were digitally recorded, transcribed, checked for accuracy and entered into NVivo™ for analysis.

**Table 2:** Interview Guide

We are interested in learning more about your perceptions of what has changed in relation to the following dimensions of palliative care in Canada since 2017. 1. What has changed in terms of palliative care education and training? a) What in your opinion has contributed to these changes and what has been the impact of these changes? b) Please describe any innovations/improvements in [framework dimension] in your area.
2. What has changed in terms of measures to support family/friend caregivers? a) What in your opinion has contributed to these changes and what has been the impact of these changes? b) Please describe any innovations/improvements in [framework dimension] in your area.
3. What has changed in terms of measures to facilitate access to palliative care? a) What in your opinion has contributed to these changes and what has been the impact of these changes? b) Please describe any innovations/improvements in [framework dimension] in your area.

The analysis was both deductive and inductive in approach. Data was first categorized deductively into categories using the national Framework priorities. Data within each category was then analyzed inductively using qualitative descriptive processes [11] and the thematic analysis techniques recommended in Interpretive Description [12]. First, to facilitate immersion in the data, two team members (BP and RC) read all of the interviews in their entirety and developed field notes and a summary of each interview. This process informed negotiating an initial set of codes, which we subsequently used to code and organize the interview data set in its entirety. On this basis, research team members (BP, RC, DW, ST) collaboratively constructed a thematic narrative account.

The quality of data analysis was supported through deep immersion by two team members in the primary data set, team members collaboratively constructing and negotiating the coding, and frequent checking back into the primary data to ensure that the findings were well grounded in the data analysis. Throughout the data collection and analysis process, rigor was further addressed through attention to epistemological integrity, representative credibility, analytic logic and interpretive authority as recommended within Interpretive Description research [12]. We also sought to demonstrate contextual awareness throughout to ensure that the interpretations we made in relation to data elements were appropriately aligned with the chronological trajectory that the changes described by different study participants represented, so that the observations and interpretations depicted in the ultimate findings report would resonate with those familiar with the Canadian palliative care context.

## Results

Here we describe thematic findings from study participants with respect to key priorities cited in the Framework [8] and the Action Plan [9], specifically educational preparation, support for caregivers, and issues of access (See Table 3).

**Table 3:** Themes across the interviews

Education for Healthcare Providers: Developing a robust and skilled health workforce to support sustainable palliative care delivery. • Embed palliative care into undergraduate preparation • Provide mentorship and ongoing learning • Be inclusive with education • Leverage the impact of palliative champions • Barriers: Failing healthcare system and practitioner burnout
Support for Family Caregivers: Understanding the unique needs of family caregivers and building greater capacity in communities to meet those needs. • Programs offered through not-for-profit • Barriers: Shortage of home support, lack of understanding of what caregivers do, policies that exclude caregivers
Access to Palliative Care: Ensuring that residents of Canada can access appropriate palliative care according to their level of need. • Nurse-centered, multi-site model • Inclusive of all care partners • Facilitated by technology • Barriers: economic, social, and system

### Education for healthcare providers

Embedding palliative content into the undergraduate preparation of healthcare providers; providing mentorship and ongoing learning opportunities; being inclusive with educational strategies; and leveraging the impact of those who become palliative champions were sub-themes relevant to education. Participants indicated that best practices in educational preparation started at the entry-to-practice level. “*We need to embed palliative care training for everyone who goes to medical school because every doctor is going to see patients die*.” (P21) A common finding across the accounts was the belief that medical and nursing entry-to-practice programs provided far too little education in palliative care. “*It was mandatory for us to learn how to birth a baby even though I might never do that in my practice. But there is no mandatory rotation in hospice or palliative care*.” (P24) This meant that the majority of palliative education needed to be provided post-graduation and participants indicated that there were robust post-graduate continuing education programs to fill this gap.

However, this continuing education often lacked mentorship and ongoing learning, key requirements for becoming competent in palliative care. “*Webinars are not equal to competency*.” (P19) Learners needed structured mentorship (e.g., fellowships, clinical rotations) in multiple contexts (e.g., home care, hospitals) that provided high quality palliative care to consolidate their knowledge and skills. “*The way our palliative service is structured*,* doctors rotate between all services*.” (P29) Participants also described locally developed communities of practice that supported such education. For example, one participant had started a popular informal gathering in which participants simply talked about their experiences of death. Another participant spoke of how integrating primary care physicians into local hospice care had provided a degree of mentorship to those physicians that might not otherwise be available in their independent practices, and how that mentorship resulted in them being more confident to manage their patients outside of hospice. “*We give them key information and then they are able to use that back in their community practice*.” (P24).

Participants universally advocated for inclusive palliative education that recognized the contributions of social workers, spiritual care practitioners, death doulas, paramedics, volunteers, home support workers, healthcare aides, and all long term care staff. “*In a palliative approach to care we need to be educating housekeepers*,* laundry*,* and dietary.*” (P10) In some provinces, the development of multi-disciplinary competencies was viewed as a major step forward in this inclusive education. “*The big thing over the last couple of years has been around [multi-disciplinary] competencies.”* (P8) Beyond these competencies, participants suggested that it was also important to construct learning trajectories that develop expertise over time. For example, nurses and physicians have access to ongoing education that facilitates evolving expertise. However, other care partners, such as personal support workers or care aides, may not have the same opportunities for ongoing learning. “*For the personal support worker there’s not a lot. Once they have gone through their training there is little education that is geared toward them*.” (P35) In raising this issue, study participants highlighted the important role that ongoing learning plays in providing a sense of satisfaction and purpose in one’s role, without which they might lose interest. “*They [healthcare aides] have told me ‘I have been working in palliative care for years. What is left for me to do and learn*?’” (P35).

As they shared their views about the importance of palliative education, some study participants specifically cited the importance of recognizing and developing palliative care champions. “*It’s far more than just a job for us*,* it’s far more than just a job.*” (P3) Participants recognized that it is often the champions who ensure sustainability. “*You need champions who will continue this conversation…not only in educating the community but in accessing palliative care*.” (P33) For example, one participant told a story of a healthcare aide whose excellent therapeutic ability with patients and families at end of life inspired an entire unit toward better care. “*She was unique. So*,* people would look to her when someone was dying. She would just do it whether she was on shift or not*.” (P34).

The major barriers to this vision of an educated and mobilized care team were what was perceived to be a failing healthcare system and practitioner burnout. Participants spoke of healthcare provider resignations and turnover which meant that their hard-won educational initiatives were wasted and they had to begin again with a new workforce. “*The turnover rate at the hospital is high. So once those nurses retire or quit there are no mentors to educate*.” (P11) Many of the participants expressed deep regret around physician and nursing shortages, both in primary and institutional care, that no amount of palliative education could overcome.

### Support for family caregivers

Even as participants described many programs offered within the not-for-profit sector to support family and other social network caregivers, they also described important barriers to realizing optimal support for these caregivers across the continuum of care contexts. Participants affiliated with hospice described a range of programs designed to support caregivers both through focusing on the caregivers themselves and through public education. These programs included day hospice, respite care, educational programs for family caregivers on how to provide hands on care, and grief and bereavement supports. Hospice societies in particular were acknowledged by participants for moving care upstream and outside of the boundaries of what would traditionally be considered palliative care in ways that would indirectly support caregivers. For example, participants described friendly visiting programs for those who were lonely and isolated, volunteer navigation programs for those who needed assistance with finding resources, programs designed to serve the needs of those living with intellectual disabilities, and grief programs for children in schools. Alongside these existing initiatives, participants spoke of how important it was for family caregivers to have some sort of guide, a consistent individual who helps them to know what is available, to provide respite, and to help them navigate the complex bureaucracy that typifies modern healthcare. “*It would be helpful to have some sort of hub or navigator role staffed by people who could help them navigate the system*,* and help us working in the system. It’s confusing and there is so much we don’t know*.” (P27) What was apparent across these interviews was that initiatives supporting family caregivers were happening primarily within the community-based, not-for-profit sector.

Participants described three main barriers to adequate caregiver support: a shortage of available in-home support, lack of understanding about what family caregivers do on a daily basis, and policies that exclude caregivers. The first barrier reflected inadequate numbers of home support workers and community nurses to assist with the day to day needs of caregivers providing care at home. The number of hours of support available to family caregivers varied significantly across the country. However, even in those jurisdictions where caregivers were allotted a sufficient number of hours of in-home support, there was no guarantee that there were enough home support workers to fulfill that commitment. *“[After COVID] Canada has now discovered what a personal support worker is and that there aren’t enough to go around.”* (P2) Participants described how these home support worker shortages often meant that family caregivers were forced to have their family member admitted to an institution for care even if they were willing to continue with care at home. Further, the time available for home care nurses to teach and support family caregivers was described as insufficient. *“[Community] nurses do not have the time and support to do that because they are pulled in so many different directions. It’s a whole other level of moral distress because they cannot give the care they recognize that families need*. (P7)

A second barrier described by participants was a lack of understanding of the work that palliative family caregivers do. They described the stigma that caregivers face because the public does not want to think about the work that they do and a lack of understanding even on the part of healthcare providers about what their day to day and long term caregiving commitment entails. “*It is heartbreaking going into homes where the person is dying. Their family members are stressed. Yes*,* we can do caregiver support but they have been stressed for five years*.” (P26) Participants described how a misunderstanding of caregiver work can result in physicians discharging patients to home without considering whether a caregiver can actually complete the requisite caregiving tasks. They explained how the needs of caregivers are rendered invisible within our systems, and hence, not addressed in the day to day care planning for patients. “*It’s terrible. There’s just so little care available for families who want to support their family member at end of life. Like*,* it’s just terrible. I think society could do more*.” (P19).

The third barrier cited were policies that excluded caregivers from professional services. For example, a physician described being unable to complete routine caregiver assessments outside of a formalized provider/patient relationship. ”*Historically*,* we had tools where we could do grief assessments and things like that on family members but now you have to have a consent from a family member because you are doing an assessment on them*.” (P8) In a related example, a healthcare provider described having to close an effective family follow-up and bereavement program when a family member launched a complaint suggesting that contacting them for follow-up entailed a breach of their privacy. Another community care provider described how, as an employee of a health region, they were not allowed to follow up with family after the death of a client. “*We see bereavement in mission statements*,* but there is not a lot of bereavement care actually being provided.*” (P33).

### Access to palliative care

Participants in this study had a common vision for what worked best in providing good access to palliative care, particularly for those living in rural areas of the country. This included a model that was nurse-centered, consult-based, multi-site, inclusive of all contributors to care, and facilitated by technology. A nurse-centered model was cited most frequently as the one that held the most promise for building palliative care capacity. “*We have developed a staffing model…and we call it a nurse-centered program*.” (P8) In this model, expert palliative care nurses are the frontline care providers who act as team coordinators and serve as the main point of access for patients and families across the care continuum. They also have the ability to triage patients and families in relation to whether they require specialized palliative care. “*They have their palliative certification and they screen every patient and liaison with the family doctor and then pull us [palliative physician] in to help with the more complex stuff*.” (P8) Participants recalled a time when such nurse-led models were the norm in community care. Community nurses who specialized in palliative care became highly knowledgeable because they worked exclusively with that population in the home. However, in multiple jurisdictions, study participants described policy changes that required nurses to become community generalists and so their palliative expertise had become diluted. *“Now I’m seeing someone that needs palliative care*,* then after that I am going to see someone with MS*,* and then someone with an ostomy*,* and someone with dementia*,* and someone with a wound. And there is no way one individual can be good at all of those things*.” (P2).

Second, a model that maximized access needed to be consult-based rather than one in which the specialist palliative physician became the primary care provider. The physicians we interviewed recommended that it was important to think carefully about optimal palliative physician population ratios and to be strategic about when and how consultations should occur. For example, the palliative approach to care, in which patients on a dying trajectory are identified and supported early, is best practice. However, this should not mean referral to a specialist palliative physician because of the impact that would have on their caseloads. “*In some places*,* as soon as you are diagnosed with metastatic disease you are referred to palliative care for symptom management*,* even if your prognosis is a year or longer. And we don’t really operate that way. It’s supposed to be within the last six months unless they have difficult symptoms. Given our caseloads that’s how we have to do it.*” (P29) Physicians indicated that being strategic about when to make the referral was important in ensuring that there was adequate capacity for care. However, this consult model had drawbacks. A number of participants expressed concerns about further fragmenting an already fragmented system. This was why the nurse-centered model was seen as important. “*We have a strong nursing component to our program so when patients are first admitted into the palliative care program*,* they’re attached to a community nurse and that nurse is always the same point of contact*.” (P29).

Third, participants described how important it was to think of access to palliative care in all sites of care including home, long term care, residential hospice, and acute medical in-patient units. This meant creating atmospheres conducive to palliative care across contexts. “*You only have one chance to die. I want a palliative person to be in a quiet environment with higher nursing ratios and soft music*,* not on a floor that is nothing but noise*.” (P25) Participants provided examples of how long term care contexts had been transformed to provide high quality care of the dying. These practices included procedures and rituals for patients, family, and staff that were both humanizing and dignifying. “*People were excited when that person was born and all their family were there. We asked ourselves*,* ‘what could we do to have that same idea for when they die*?’” (P34).

Fourth, participants advocated for improving access through meaningful involvement of the entire circle of care including social workers, spiritual care providers, death doulas, volunteers, paramedics, and ultimately the public. “*We are seeing palliative care as more of a circle of support as opposed to a medical program*.” (P26) Almost every participant remarked on having witnessed major gains in public awareness about palliative care. “*We are in a time period where people are less death denying than ever before*.” (P24) However, they also acknowledged that the practical processes through which to engage the public and the full circle of care were not necessarily available. There were exceptions such as the implementation of whole community palliative rounds in one jurisdiction in which all care team members, including paid and volunteer carers, were brought together regularly to communicate about how to best serve patient needs. An innovation described by participants was the involvement of paramedics in the palliative care team. This model, which is expanding across Canada, draws upon paramedics to provide urgent in-home palliative treatment. One paramedic participant who had been involved with this innovation indicated the scope of the change it represents. “*This is a massive change in relation to my profession. So*,* thousands of paramedics now get palliative care training when before there was nothing.*” (P18) From the perspective of many of our study participants, this seems a feasible and pragmatic solution to the challenges of providing family caregiver support and symptom management outside of “regular” hours.

Finally, participants described the emerging role of technology in facilitating better communication among palliative team members, and therefore creating a higher quality palliative care for patients. Communication among providers was widely recognized as a longstanding problem. “*It’s the lack of communication across all sectors. It doesn’t serve the patient well*.” (P35) As an example of a technological innovation, one participant described a secure phone-based application that allowed them to consult with one another in real time. “*You can actually use names and bring as many people into the conversation as you need to and it’s done really quickly*.” (P35) Others described the increasing use of virtual consultations. However, although virtual consultations enabled healthcare providers to stay in closer touch with patients and family, some participants wondered whether the virtual context led family caregivers and patients to downplay developing problems. For many, in-person interactions seemed the optimal context in which healthcare providers were able to identify developing problems and intervene before they became crises.

Barriers to palliative care access described by participants were economic, social and system factors. Participants, particularly those in the hospice, long term care, and community sectors, described the effects of privatization on a system that was already struggling. They suggested that, once profit became a primary motivator, there was too much temptation to create a lean system that was unable to accommodate the changing needs of a vulnerable population. In long term care and community settings this was manifest in low staffing numbers, heavy workloads, and insufficient preparation for healthcare providers to do their assigned role well. “*We talk about person-centered care but really the care is about whoever gets the contract. It’s all business*,* right? I don’t think I realized to what degree medicine is influenced by business until the last few years*,* which is kind of disappointing to say the least*,* and it’s morally distressing*. (P19) Further, despite the important services that hospice societies provide for patients and family caregivers, they struggled to sustain funding for that work. “*We’re too small of a hospice to do much outside of fundraising. We struggle to keep our lights on*.” (P5).

Social barriers often arose as a result of these economic barriers. For example, residential hospices that were built through community fund-raising but then operated through public and/or private partnerships demonstrated unique social tensions. Participants contrasted the philanthropic spirit that had built the facility with the for-profit business model that was adopted to run it. As one participant described it: “*It’s a bit like being occupied by a foreign army. They are here in our building that we paid for and they’re operating a program and telling us what to do inside that building*.” (P4) Other social barriers included inequitable access for populations made marginal within our society. “*So*,* the system seems to be built with a certain person in mind*,* a fairly financially well off*,* medically literate*,* educated*,* English-speaking patient*.” (P27) Persons from visible minority communities for whom English was a second language were particularly at risk for poor access to palliative care. “*There are people who are racialized who don’t get good palliative care because they don’t know how to ask for services*.” (P2) They recognized that inhabitants of rural and remote communities in Canada are also underserved. “*One of the hardest things I see as a physician is knowing that my patient who lives rurally will not get the same access to palliative care as my patient who lives in town*.” (P21).

System level barriers described by our study participants were twofold: the siloed nature of healthcare and the variability in services even within health regions. Participants described how the siloed nature of healthcare is exacerbated when the circle of care crosses both professional and community not-for-profit and for-profit sectors. “*The [private] long term care homes have an objective and that is to make money. And so*,* you are not necessarily getting someone that has a lot of experience and you’re getting a lot of fragmented care*.” (P2) Participants from hospice described the silos between volunteer and professional services, specifically describing how little healthcare providers often knew about voluntary contributions to a palliative approach to care. Without stronger integration across these sectors, patients had difficulty navigating the system and gaining the information and support they needed. This made for difficult patient transitions across healthcare contexts. One participant likened these transitions to patients “*falling off a cliff*.” (P18) Finally, study participants confirmed how powerfully access to palliative care services can be affected depending on which healthcare region the patient resides within. “*The line between the health regions crosses a particular street*,* someone who lives on one side of the street is going to get different healthcare than someone who lives on the other side*.” (P21).

## Discussion

Findings from 35 palliative care stakeholders provided important insights both about how to provide better access to palliative care, and perhaps more importantly, about the practical barriers that were hindering the realization of that vision. Participants described three key approaches that could be used by decision-makers to help realize the goals set forward in the Framework [8] and in the Action Plan [9] for palliative care in Canada: developing competent palliative champions, optimizing palliative care organization in the face of workforce shortages, and supporting family caregivers strategically.

### Developing competent palliative champions

Participants believed that comprehensive palliative education has yet to be properly integrated into undergraduate health professional preparation, a finding that has been supported in other studies. A survey of undergraduate medical education in Canada indicated that rotations in palliative care were only required in two of the schools surveyed, and in the 2015/2016 graduating class only about 30% of undergraduates had completed clinical rotations in palliative care. This percentage was an improvement over 2011/2012, where only 13.6% had palliative clinical rotations [13]. Ongoing surveys will be needed to confirm whether that trend has continued, or whether recent challenges in the healthcare system may have compromised it. Likewise, a survey of undergraduate nursing education programs in Canada (*n* = 24) indicated that palliative and end of life competencies were addressed in all programs but most commonly the knowledge was threaded throughout existing courses, therefore difficult to quantify with any accuracy [14]. In their five-year progress report on the Framework and Action Plan, Health Canada similarly highlighted the need for increased education and mentorship for healthcare provider students [15]. 

Participants further recognized that theoretical education was simply insufficient to create confident palliative care practitioners and champions. Relying solely on education to solve long-standing issues related to palliative has frequently been acknowledged in the literature as a problem [16, 17]. The difficulties in definitional aspects of palliative care, it’s multi-disciplinary and holistic nature, and the range of settings within which it is provided make for a complex educational process [18]. Therefore, goals associated with educating practitioners cannot be considered apart from the contextual factors that support or inhibit practitioners from applying what they have learned. For example, a great deal of evidence has been generated in the last few years about implementing a palliative approach in long term care. We know that education alone cannot ensure a palliative approach to care in environments characterized by high acuity, chronic staffing shortages, high patient to staff ratios, and little time available for patient psychosocial support [19]. Adaptations for various contexts and needs will have to be considered to ensure that the outcomes of such education include confidence and a commitment to the advantages of a palliative approach to care.

We suggest that the perceived relationship between palliative education and the development of palliative champions seems an important finding. A number of the participants described their passion for this care and the sense of calling that had persisted over a career trajectory. This importance of palliative champions has been identified in the literature [20–22]. Although the role of the champion is not always well defined [23] and cannot always be mapped to improved outcomes [24], this would suggest that it is not just about educating those involved in palliative care but also about using that education to identify those who might become the champions for care. On that basis, we would need to develop them further into clearly defined palliative leadership positions that can be sustained over time.

### Optimizing palliative care organization

A theme across the data was the healthcare workforce shortages and its subsequent impact on palliative care. The shortage of family physicians, home care nurses, and home support nurses was cited as particularly acute. In 2019, 14.5% of Canadians did not have access to a regular healthcare provider [25]. In 2022, the nursing shortage in Canada has been referred to as a “national emergency” with nurses accounting for 45% of all job vacancies within health [26]. In light of these shortages, the goal of a palliative approach to care embedded within the primary care system will be difficult to achieve unless this underlying problem is addressed.

Nevertheless, participants in this study had a shared vision of a model of care designed to maximize palliative care capacity. Physicians we interviewed spoke of the importance of having an expert palliative nurse as the point of contact for patients and family who could then coordinate and connect the multi-disciplinary team. This nurse would perform an important role in identifying which patients required specialized palliative care and which could be managed within the primary care system. This would mean that specialized palliative physician time could be used for maximum impact. This finding is particularly important for rural areas, where physicians who have advanced preparation in palliative care often also have their own family practices [27, 28]. However, such a nurse-centered model may be difficult to implement in rural settings simply because there may be insufficient numbers of palliative patients to justify a dedicated palliative nurse. Research in rural areas in Canada has demonstrated the importance of expert palliative nurses in building capacity for care and the importance of using them strategically [29]. This model differs from some current work force policies that require every community nurse to work as a generalist. On the basis of our findings, it would seem that these palliative champion nurses might well take on other patients in community, but their workloads may require flexibility so that those nurses who have an interest and expertise in palliative care are deployed to care for that population as needed, and can follow them across contexts in a consult model.

The other necessary component for excellent palliative care access according to our study participants is seamless and timely communication among team members. Whole community rounds are an important innovation in connecting the palliative team across rural and urban contexts [30]. The use of technology to communicate in new ways was also highlighted by participants as particularly important, an innovation that developed rapidly through COVID. For example, the development of a phone-based application that allowed rapid and private communication between team members was seen as particularly helpful.

### Supporting family caregivers strategically

In our recent survey of stakeholder perceptions of improvements in palliative care, the aspect in which the least improvements had happened was in support for family caregivers, including respite, bereavement services, and in-home support [10]. Health Canada similarly identified the importance of enhanced support for family caregivers in the home as an important action plan based upon their five-year review of progress on the Framework [15]. A recent scoping review on support for informal caregivers identified the importance of psych-social-spiritual, financial, educational, and indirect patient support for these important care partners [31]. Participants in this study who worked with not-for profit hospice societies described a wealth of programs to provide practical help to family caregivers and to support them into bereavement. However, these programs are often limited by capacity. For example, a recent study of hospice societies in British Columbia indicated that 83% of these societies served caregivers and 88% served grieving family members or friends. In 2018, the average number of clients served per month over the 47 organizations reporting was 9,790. Despite these substantial contributions, they reported the majority of their revenue coming from grants, donations, and fund raising. Their two greatest needs were for education and training (65%) and fund development (56%) [32]. This is the essence of the challenge highlighted by the Palliative Care Coalition of Canada when they suggested that too much rests upon the volunteer sector [6]. Strategic support of selected programs aimed at family caregivers would help to boost capacity in the not-for-profit sector. Further, there is a clear need for policy work to ensure that family caregivers are seen as relevant partners in care without having to become patients. The Canadian National Seniors Strategy recommends conducting individualized needs assessments for all caregivers [33]. Finding ways to conduct these assessments as a part of routine care seems of critical importance. As family physicians are often the primary point of care for family caregivers, removing policy barriers to conducting those individualized needs assessment should be an important consideration.

### Strengths and limitations

There are limitations that should be considered when evaluating the results of this study. We recognize that, although the sample represented considerable diversity of health profession, role in palliative care, geographic location and context as well as extensive palliative care experience, the number of stakeholders who participated represented a relatively modest sampling of all possible perspectives. Further, because the interviews were conducted while COVID was still impacting healthcare environments, the findings must be viewed within that context. Despite these limitations, a strength of the study was that those who participated demonstrated a clear vision, from multiple angles, for the developmental of palliative care in Canada. In this way, they lend credence to the vision and ongoing action priorities articulated in Health Canada’s five-year report on the Framework and Action Plan [15] and provide many relevant insights with respect to the central role that nursing can play in realizing this vision.

## Conclusion

Findings from this study provided important insight with respect to strategic and policy oriented approaches to enhancing palliative care in Canada. Although it is important to ensure robust palliative care education for healthcare students, it is equally important to ensure that workplace contexts allow healthcare professionals to enact high quality palliative care. In particular, our findings demonstrate clear mechanisms through which enhancing palliative care access can be achieved through advancement of more robust and strategic nursing roles. Although this study did not explicitly feature reflections on access to palliative care for populations on the margins, it seems reasonable to assume that their access to palliative services could also be enhanced by strengthened capacity within the groups of nurses whose practice is closest to their care. Some of these findings, such as how to organize palliative care and develop champions, seem eminently achievable within current health delivery and educational systems. Other findings, such as acute workforce shortages, will require longer term and more comprehensive solutions.

How to further enhance support for family caregivers, who are arguably the backbone of the palliative care system, seems less clear. Providing sustainable funding to organizations that provide innovative programs for family caregivers seems an important policy direction. However, the greatest challenge constitutes deciding where they fit into the overall system. On the basis of what we have learned through this study, it is apparent that a strategic and policy-oriented approach is required so that the capacities of both the paid healthcare system and the not-for-profit system are leveraged to close gaps for these essential care providers. Once such mechanisms have been worked out, nursing seems ideally positioned to play a meaningful role in both supporting patients and families and expanding access to palliative care approaches across all of the relevant health care sectors.

## Data Availability

The datasets generated during and/or analyzed during the current study are available from the corresponding author on reasonable request.
